# Deep learning–based approaches for weed detection in crops

**DOI:** 10.3389/fpls.2025.1746406

**Published:** 2026-01-09

**Authors:** Hua Zhao, Yan Wang

**Affiliations:** School of Mechanical Engineering, Jiangsu Ocean University, Lianyungang, China

**Keywords:** deep learning, image segmentation, image classification, object detection, weed detection

## Abstract

Deep learning has become a transformative technology for modern weed detection, offering significant advantages over traditional machine vision in robustness, scalability, and recognition accuracy. This review provides a comprehensive synthesis of recent progress in deep learning-based weed detection, with a focus on three major model families: object detection, image segmentation, and image classification. For each category, representative architectures, key algorithmic features, and typical agricultural application scenarios are summarized and compared. The strengths and limitations of these approaches—particularly in terms of spatial localization, pixel-level delineation, computational efficiency, and model generalization—are critically analyzed. In addition, major challenges such as dataset scarcity, annotation cost, variability in weed morphology, and real-time deployment constraints are discussed, along with emerging solutions including crop-based indirect detection, semi-supervised learning, and model–actuator integration. This review highlights future opportunities toward scalable, data-efficient, and precision-integrated weed management, offering guidance for the development of next-generation intelligent weeding systems.

## 1 Introduction

Weeds remain one of the most damaging biotic constraints to global crop production, imposing substantial yield and quality losses through intense competition for light, water, nutrients, and space (Kaur et al., 2018). Their rapid reproduction, strong ecological adaptability, and ability to thrive under diverse environmental conditions often enable them to outcompete crops, threatening agricultural sustainability and food security. Studies have shown that uncontrolled weed infestations can significantly reduce crop yield, depending on species composition and management practices (Vilà et al., 2021). Moreover, the proliferation of herbicide-resistant weed biotypes and the increasing variability of weed populations across regions further complicate effective control (Shaner, 1995). Beyond direct yield impacts, weeds also increase production costs, hinder harvesting operations, and contribute to long-term ecological risks (Bairambekov et al., 2016; Wicks et al., 2017). Therefore, efficient and timely weed management is essential for stabilizing crop production and ensuring the long-term resilience of agricultural systems.

With the shift toward precision agriculture, weed control has progressively evolved from uniform field-scale treatment to spatially targeted and environmentally conscious management. Mechanical weeding in inter-row areas requires reliable visual perception to avoid damaging crop stems, while intra-row mechanical systems depend heavily on precise localization to remove weeds growing near crops (Balas et al., 2022). Similarly, site-specific herbicide application relies on accurate identification of weed presence, species, and distribution patterns to minimize chemical inputs while maintaining high control efficiency. Conventional broadcast spraying wastes substantial amounts of herbicide on non-target surfaces and contributes to ecological contamination, whereas precision spraying systems can significantly reduce chemical use when supported by accurate weed detection (Reddy and James, 2018). Consequently, robust weed detection has become a foundational requirement for intelligent weeding robots, autonomous tractors, and UAV-based prescription mapping (Xu et al., 2025; Huang et al., 2018). The increasing demand for high-resolution, real-time, and scalable weed perception underscores the necessity of advanced computational approaches capable of operating in heterogeneous and complex field environments.

The rapid development of deep learning, particularly convolutional neural networks (CNNs) and Transformer-based architectures, has fundamentally reshaped the landscape of computer vision. These models have achieved breakthroughs in medical imaging (Kim et al., 2019), autonomous driving (Grigorescu et al., 2020), remote sensing (Dou et al., 2021), industrial inspection (Glaeser et al., 2021), and robotics (Chen et al., 2020) by enabling hierarchical feature learning and delivering high robustness under substantial variation in lighting, texture, scale, and occlusion. In agriculture, deep learning has demonstrated remarkable success in tasks such as disease diagnosis, fruit detection, yield prediction, canopy segmentation, and crop phenotyping, outperforming traditional machine vision and classical machine learning techniques (Deng et al., 2021; Wang et al., 2022; Chu and Yu, 2020; Atkins et al., 2025). Its ability to automatically extract discriminative features from raw images without relying on hand-crafted descriptors makes it especially valuable in field environments characterized by non-uniform illumination, dynamic backgrounds, and biological diversity (Argunşah et al., 2025). These cross-domain achievements illustrate the strong potential of deep learning to serve as the core enabling technology for next-generation intelligent weed management systems.

Traditional weed detection methods primarily rely on manually engineered features derived from color indices (Rehman et al., 2018), texture descriptors (Pan et al., 2016), or spectral characteristics (Ajamian et al., 2021). Although effective in controlled environments, their performance deteriorates under variable illumination, soil clutter, occlusion by crop leaves, morphological variation in weeds, and changes in crop growth stages (Zhou et al., 2025). Deep learning overcomes these limitations by learning multi-scale, high-level semantic representations that capture complex spatial and contextual patterns, enabling more robust differentiation between crops and weeds. Object detection models can localize weeds with bounding boxes, segmentation networks can delineate weed shapes at the pixel level, and lightweight classification networks can distinguish weed species or growth stages with high accuracy (Rai and Sun, 2024). Moreover, deep learning models can be fine-tuned, transferred across regions, or combined with multispectral, hyperspectral, or depth imagery to enhance generalization (Liu et al., 2021). These advantages make deep learning particularly well suited for real-time weed perception in precision agriculture while providing a scalable framework adaptable to diverse sensing platforms and field conditions (Li et al., 2024b).

Given the rapid expansion of deep learning applications in weed detection and the increasing availability of field imagery from UAVs, ground robots, and intelligent implements, a comprehensive and updated review is needed to synthesize existing progress, identify limitations, and outline future opportunities. This review summarizes recent advances in deep learning-based weed detection with an emphasis on three major frameworks—object detection, image segmentation, and image classification. For each category, representative network architectures, application scenarios, and performance metrics are analyzed and compared. The advantages, limitations, and trade-offs among these approaches are discussed, providing guidance for selecting suitable models under different field conditions and computational constraints. Finally, the review outlines key challenges and future research directions toward scalable, robust, and integrated intelligent weed management. The remainder of this paper is organized as follows. Section 2 reviews deep learning-based methods for weed detection, including object detection, image segmentation, and image classification. Section 3 discusses the main challenges related to model design, dataset construction, and system deployment, and outlines potential solutions and research directions. Section 4 concludes the paper.

## 2 Deep learning-based methods for weed detection

**Figure 1:**
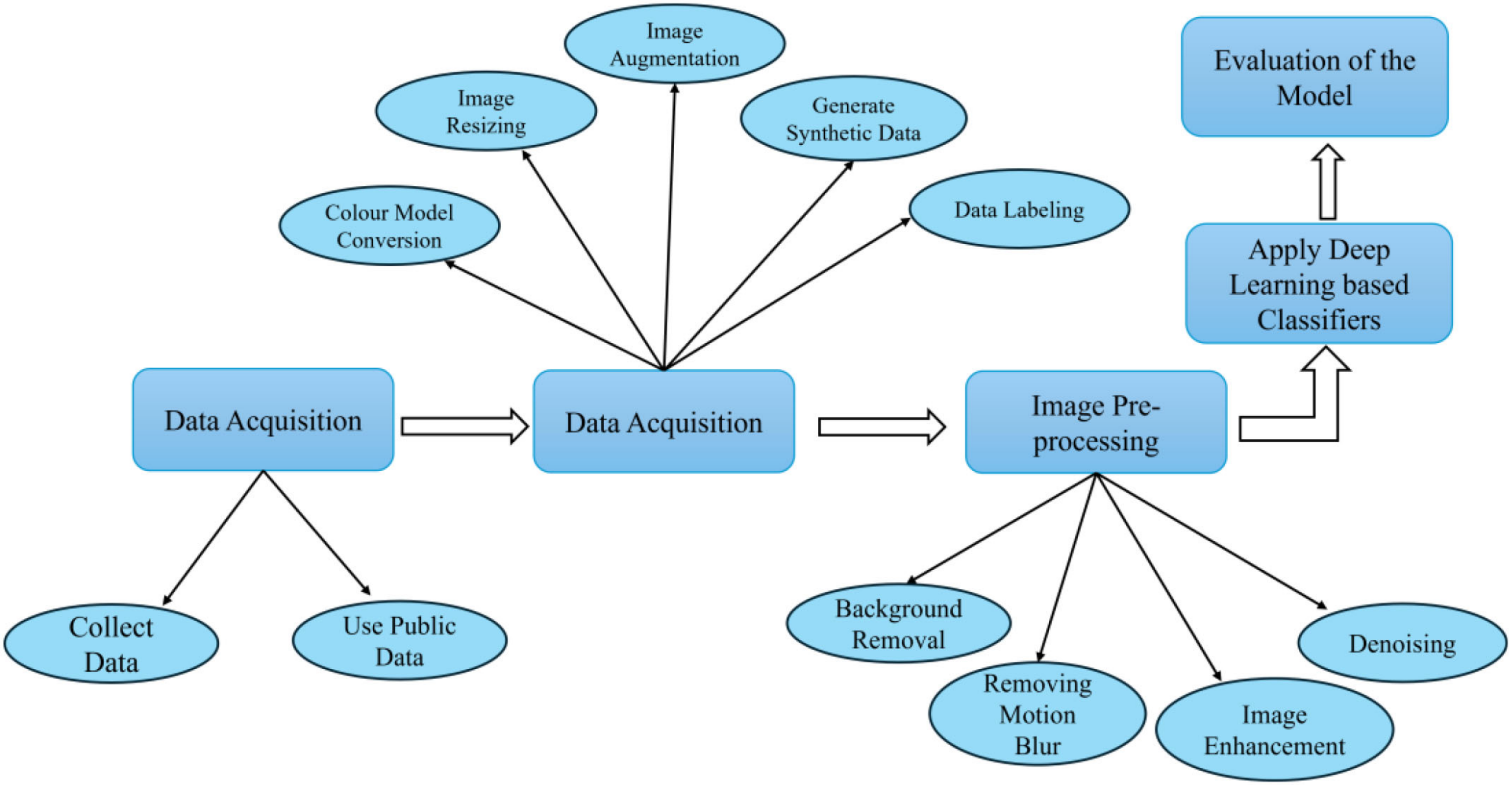
Overview of deep learning-based weed detection process (Hasan et al., 2021).

Deep learning has fundamentally transformed computer vision by enabling neural networks to automatically learn hierarchical feature representations from raw image data, avoiding the need for handcrafted feature engineering required in traditional machine learning approaches (Lecun et al., 2015). In the context of weed detection, deep learning models have shown strong robustness against variations in illumination, occlusion, weed density, and background complexity, making them highly suitable for complex agricultural environments (Teimouri et al., 2022). A typical deep learning-based weed detection workflow consists of data collection, dataset annotation, image preprocessing, model training, inference, and evaluation (**Figure 1**) (Hasan et al., 2021). According to their learning objectives and output structures, deep learning methods for visual weed detection can be broadly grouped into three categories—object detection, image segmentation, and image classification. These model families form the algorithmic foundation for subsequent research and application in intelligent and precise weed management.

### 2.1 Categories of deep learning models

Deep learning models for visual perception are commonly instantiated as object detection, image segmentation, or image classification networks, each characterized by distinct architectures, learning objectives, and output representations. These model categories form the algorithmic foundation for a wide range of computer vision tasks in both natural and structured environments.

Object detection models aim to simultaneously localize and classify objects within an image by predicting bounding boxes and corresponding category labels. Two-stage detectors, such as Faster R-CNN (Ren et al., 2017), generate region proposals before refining them through dedicated classification and regression heads, providing high accuracy at the cost of computational complexity. In contrast, one-stage detectors, including SSD (Liu et al., 2016), YOLO (Redmon et al., 2015), and RetinaNet, eliminate the proposal step and directly perform dense regression on predefined anchor boxes or anchor-free keypoints, enabling significantly faster inference. Modern object detectors incorporate feature pyramid networks (FPN), multi-scale feature aggregation, attention mechanisms, and Transformer-based modules to enhance small-object detection and improve robustness across varying image resolutions (Lu et al., 2025; Zhao et al., 2023; Lin et al., 2022).

Image segmentation models focus on pixel-level classification by assigning a semantic label to each pixel in an image. Semantic segmentation architectures such as FCN and U-Net adopt encoder–decoder designs, where the encoder extracts progressively abstract features and the decoder reconstructs spatial detail through upsampling and skip connections (Ates et al., 2023; Agarwal et al., 2023). More advanced models like DeepLab series integrate atrous convolutions and multi-scale context aggregation (ASPP), while Transformer-based architectures leverage long-range dependencies for improved structural consistency (Sharma et al., 2025). Instance segmentation models, exemplified by Mask R-CNN, further distinguish individual object instances by combining detection heads with dedicated mask prediction branches (He et al., 2020). These models are widely used when fine-grained spatial structures or object shapes are critical.

Image classification models seek to assign a single category label to an image or image patch. Classical convolutional neural networks such as AlexNet (Krizhevsky et al., 2017), VGG (Simonyan and Zisserman, 2014), GoogLeNet (Szegedy et al., 2014), and ResNet (He et al., 2015) pioneered deep hierarchical feature extraction, while later architectures—including DenseNet (Huang et al., 2016), MobileNet (Howard et al., 2017), EfficientNet (Tan and Le, 2019), and RegNet (Xu et al., 2021)—optimized parameter efficiency, representational capacity, and deployment feasibility. Recently, Vision Transformers (ViT) and hybrid CNN–Transformer models have demonstrated strong performance by modeling global spatial relationships through self-attention mechanisms (Dosovitskiy et al., 2020; Li et al., 2024a). Classification networks form the backbone of numerous visual recognition tasks and can be adapted for fine-grained species identification, hierarchical categorization, or lightweight edge deployment.

### 2.2 Object detection methods for weed detection

**Figure 2:**
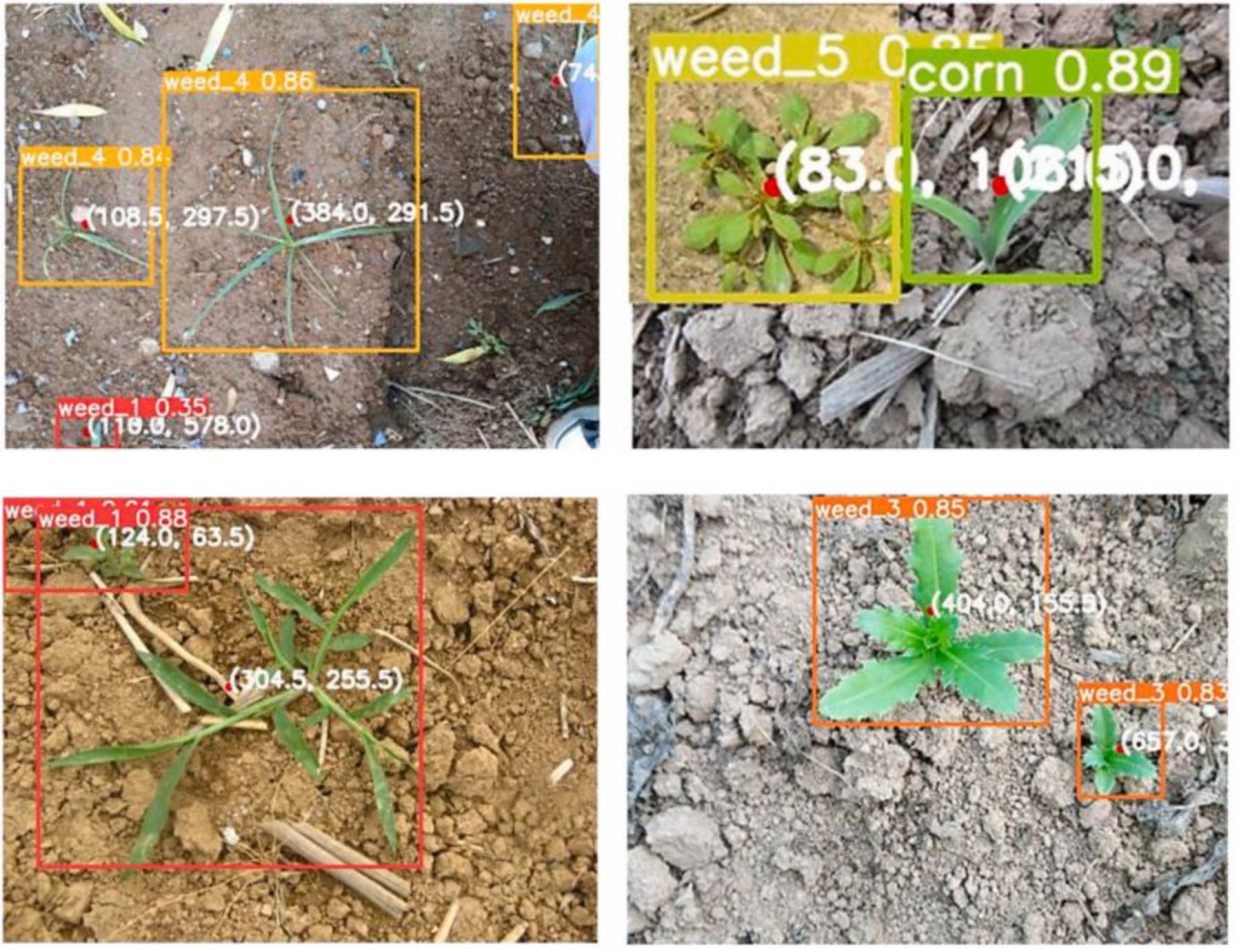
Detected objects in sample Images (Sun et al., 2024).

In the context of weed detection, the primary goal of object detection models is to identify weed plants in images and accurately localize them through bounding boxes (**Figure 2**) (Darbyshire et al., 2023; Sun et al., 2024). Directly applying general object detection networks—such as YOLO and Faster R-CNN—has become one of the most widely adopted approaches in weed detection. For example, Deng et al. (2023) proposed a YOLOX-based weed detection method for paddy fields, in which CSPDarknet combined with FPN was used for multi-scale feature fusion. Among multiple compared models, YOLOX-tiny achieved the highest accuracy and lowest computational cost in dense small-object scenarios during the seedling stage, making it suitable for deployment on embedded agricultural devices for precision spraying. In addition, Darbyshire et al. (2023) conducted a comprehensive investigation on weed detection from the perspective of precision spraying. Their study evaluated detection accuracy and spraying performance using two independent datasets, multiple image resolutions, and several state-of-the-art object detection algorithms. To realistically represent precision spraying outcomes, a simplified spraying model was introduced, along with two key metrics—”weed coverage” and “sprayed area”—to jointly assess weed hit rate and herbicide use efficiency. The results demonstrated that, when using state-of-the-art visual detection methods, spraying only 30% of the field area could cover 93% of weeds, highlighting the potential of deep-learning-based precision spraying to substantially reduce herbicide consumption and minimize environmental impact.

Based on the direct applicability of object detection for in-field weed monitoring, researchers have developed and optimized a variety of deep-learning-based weed detection models. **Table 1** lists representative models, highlighting their network architectures, target objects, and performance metrics in typical agricultural scenarios.

**Table 1:** Representative deep-learning-based object detection models for in-field weed detection.

Reference	Target(s)	Detection model(s)	Results
(Li et al., 2024b)	Winter wheat weed (3W dataset)	YOLOv8_NLB	Achieved an mAP of 88.3%
(Etienne et al., 2021)	Monocot and dicot weed species in corn and soybean fields	YOLOv3	Using training set 4 (images at 10 m AGL): AP @ IoU 0.25 – Monocots: 91.48%, Dicots: 86.13%; AP @ IoU 0.5 – Monocots: 63.37%, Dicots: 45.13%
(Deng et al., 2023)	Rice field weeds at seedling stage (small and dense targets)	YOLOX-tiny (compared with YOLOv3, YOLOv4-tiny, YOLOv5-s, SSD, YOLOX-s, YOLOX-m, YOLOX-nano)	mAP: 0.980, F1: 0.95, Recall: 0.983; intermediate variable memory: 259.62 MB; suitable for embedded agricultural devices; improved detection of occluded and dense small weed targets in rice seedlings
(Yang et al., 2025)	Weeds and bok choy in UAV images (Brassica rapa subsp. chinensis)	WeedDETR (RepCBNet backbone + Feature Complementary Fusion Module FCFM + Zoom Loss)	AP0.5: Weeds 73.9%, Bok choy 91.8%; inference speed 76.28 FPS; compared with YOLOv5-L, YOLOv6-M, and YOLOv8-L, weeds AP0.5 increased by 3.5%, 6.3%, and 3.6%, FPS increased by 14.9%, 12.9%, and 1.4%; enables efficient end-to-end small-target weed detection
(Xu et al., 2024)	Gramineae weeds in wheat fields	WeedsNet (dual-path feature extraction network + multimodal fusion + attention mechanism)	Accuracy in natural wheat fields: 62.3%; detection speed per image ~0.5 s; depth information complements RGB features, enhancing detection accuracy
(Yu et al., 2019)	Bermuda grass weeds and other broadleaf weeds	VGGNet, GoogLeNet, DetectNet/VGGNet, GoogLeNet, DetectNet	VGGNet F_1_ > 0.95 for Hydrocotyle spp., Hedyotis cormybosa, Richardia scabra; DetectNet F_1_ > 0.99 for Poa annua and weeds in dormant Bermuda grass; reliable detection across mowing heights and surface conditions

Although object detection allows direct identification of visible weeds in images and provides information on their location and abundance, certain limitations remain in practical applications. The advantages of using object detection networks lie in their ability to rapidly process large-scale field images and directly obtain the spatial distribution of weeds, enabling automated monitoring. Deep learning networks can extract multi-scale features of weeds from complex field backgrounds, thereby improving detection accuracy. Furthermore, some lightweight models, such as YOLOX-tiny and MobileNet-SSD, can achieve real-time inference on embedded devices, offering feasible deployment solutions for UAVs and field robots.

On the other hand, the limitations of employing object detection networks for weed detection are also significant. Detection performance is highly affected by variations in illumination, crop growth stages, weed density, and occlusion by crops, with small-sized weed plants particularly prone to being missed or misclassified, resulting in reduced model accuracy. Furthermore, the detectable weed species are limited to those present in the training dataset, leading to poor generalization across new regions or different seasonal conditions. In addition, achieving high-precision detection requires a large volume of annotated data, the production of which is costly and time-consuming, thereby constraining the widespread application of these models in practical precision agriculture.

### 2.3 Image segmentation methods for weed detection

**Figure 3:**
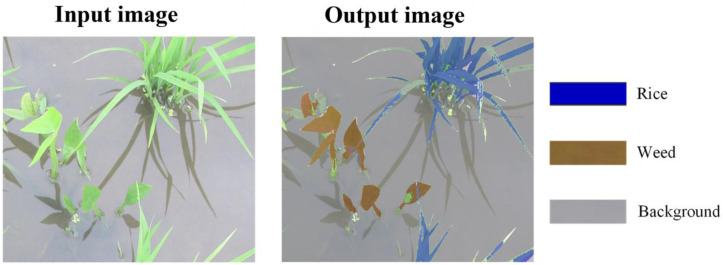
Image segmentation results for weed detection in paddy fields (Ma et al., 2019).

Image segmentation is a fundamental task in machine vision and includes semantic segmentation and instance segmentation. Semantic segmentation assigns a class label to every pixel in an image, thereby delineating precise object regions; however, it does not distinguish between different instances of the same class. In contrast, instance segmentation performs both pixel-level classification and object localization, providing accurate object shapes while differentiating multiple instances within the same category (Nistor et al., 2020). Unlike object detection, which only predicts bounding boxes, image segmentation provides pixel-level information and can capture precise object contours (Minaee et al., 2021). Image segmentation is widely used in a variety of computer vision applications, including medical imaging (Hoorali et al., 2022), autonomous driving (Papadeas et al., 2021), and agricultural analysis of crops and weeds (Fawakherji et al., 2021). **Figure 3** illustrates the segmentation network results for weed detection in paddy fields.

Compared with object detection, deep-learning-based weed segmentation provides pixel-level delineation of weed distributions and shapes, which is particularly beneficial when weeds grow in close proximity to crops or are partially occluded. This fine-grained representation offers more precise guidance for precision agriculture. Xu et al. (2023) proposed a weed detection method that integrates visible light color indices with an encoder–decoder-based instance segmentation model. The color indices enhanced the contrast between vegetation and soil, mitigating the effects of illumination variation and background interference. Meanwhile, the combination of ResNet101_v and DSASPP improved multi-scale semantic feature extraction and boundary segmentation. Field experiments demonstrated high accuracy (Acc = 0.905, IoU = 0.959), and the overall performance surpassed or matched that of Deeplabv3+, Deeplabv3, FCN, U-Net, FastFCN, Swin Transformer, and Vision Transformer, confirming its potential for application in precision weed control.

To illustrate the progress of deep learning-based weed segmentation, **Table 2** lists representative segmentation models reported in recent years, highlighting the target of detection, the segmentation network employed, and performance metrics in typical agricultural scenarios. This facilitates a clear comparison of different models in accurately depicting weed distribution and shape.

**Table 2:** Representative deep-learning-based image segmentation models for in-field weed detection.

Reference	Target(s)	Segmentation model(s)	Results
(Rai and Sun, 2024)	UAS remote sensing weed images	Single-stage deep learning architecture (C3/C3x backbone + ProtoNet head)	Using C4 data augmentation, achieved 85.4% bbox AP and 82.8% mask AP on GPU; 79.1% bbox AP and 77% mask AP on edge device (AGX Xavier); enables real-time point-spray detection and weed masking estimation
(Genze et al., 2022)	Weeds in sorghum fields (UAV images)	U-Net-like architecture with ResNet-34 backbone	F_1_ score > 89% on hold-out test set; accurate prediction of general plant shapes with most misclassifications at plant boundaries; capable of detecting in-row and partially occluded weeds; demonstrates strong generalization under challenging capture conditions
(Wang et al., 2021)	Solanum rostratum Dunal (UAV images)	DeepSolanum-Net (U-Net-based CNN)	Pixel-wise detection of Solanum rostratum Dunal with 89.95% precision and 90.3% recall; coverage and ground area calculated from segmentation results
(Das and Bais, 2021)	Weeds, damaged rapeseed leaves, crops, background (images collected under natural conditions)	DeepVeg (deep learning semantic segmentation model)	Mean IoU > 0.76 and accuracy > 0.97 for four-class segmentation; robust to unannotated, newly grown weeds and rapeseed; can differentiate circular-like weeds and seeds with limited training data
(Kong et al., 2024a)	Corn field weeds	Enhanced YOLO-based segmentation network (with cross-attention module)	Achieved mean IoU50 of 90.9%, accuracy improved by 5.9%, GFLOPs reduced by 15.56%, demonstrating suitability for deployment in resource-constrained environments

**Table 2** presents representative deep learning-based weed segmentation models reported in recent years, and the advantages and limitations of these approaches warrant further discussion. Deep learning-based segmentation methods can achieve pixel-level accuracy, enabling precise delineation of weed distribution and morphology, which is particularly important in complex field environments where weeds grow in close proximity to crops or are partially occluded. In addition, their multi-scale feature extraction capability allows these models to handle weeds of varying sizes, and the resulting segmentation maps can be directly applied to downstream tasks such as precision spraying, yield estimation, or weed density analysis, thereby enhancing the level of agricultural intelligence. Moreover, pixel-level segmentation reduces human judgment bias and improves detection reliability.

However, these methods face several challenges in practical applications. First, the high diversity and complex morphology of weeds make them heavily dependent on large-scale pixel-level annotated datasets, which are costly and labor-intensive to acquire. Second, achieving high-precision pixel-level segmentation often increases computational demands, posing greater challenges for real-time inference. Third, environmental factors such as illumination variation, shadows, and color similarity between weeds and crops can negatively affect segmentation accuracy, and the generalization ability of models across different regions, crop types, or seasons may be limited, often requiring fine-tuning or transfer learning. In summary, while deep learning-based weed segmentation provides highly precise information, trade-offs in terms of data requirements, computational cost, and generalization need to be carefully considered.

### 2.4 Image classification methods for weed detection

The advantage of using deep learning for image classification lies in its ability to automatically learn features from images through end-to-end training, enabling efficient and accurate automated image categorization. In recent years, machine learning, particularly deep learning, has been widely applied to various image classification tasks across fields such as medicine (Lei and Li, 2025; Xu et al., 2022), sports analysis (Wang et al., 2018), agriculture (Peng and Wang, 2021), and aerospace (Liu et al., 2020), significantly enhancing both model generalization and classification performance.

Based on the aforementioned advances, the application of deep learning-based classification models in weed detection has been gradually increasing. By automatically extracting discriminative features from crop and weed images, these models can classify vegetation at both the species and growth stage levels, enabling rapid and accurate weed identification in complex field environments. Such approaches reduce the reliance on manually designed features inherent in traditional machine learning and facilitate high-quality analysis in large-scale agricultural scenarios, providing strong support for precision weed management and site-specific herbicide application. For example, Almalky and Ahmed (2023) employed UAV-acquired datasets of four growth stages of Consolida regalis and applied deep learning models including YOLOv5, RetinaNet, and Faster R-CNN. They successfully achieved real-time weed detection and growth stage classification, with the YOLOv5-small model achieving a maximum recall of 0.794, and RetinaNet (with a ResNet-101-FPN backbone) attaining an average precision of 87.457%, demonstrating high accuracy and precision.

However, since image classification tasks can only assign an image to a certain category and cannot determine the exact location of weeds within the image, using classification models for weed detection often cannot directly localize weeds and requires additional detection methods. For example, Jin et al. (2022) proposed a grid-based approach, in which each image is divided into sub-images (grid cells), and the presence of weeds in each cell is determined to localize weeds within the image. Additionally, they evaluated several convolutional neural network architectures, including DenseNet, EfficientNetV2, ResNet, RegNet, and VGGNet, for weed detection and classification tasks. Specifically, they conducted (i) a multi-class classification task to distinguish between turfgrass and multiple weed species, and (ii) a binary classification task to differentiate weeds from turfgrass, thereby assessing the feasibility and effectiveness of different networks for accurate weed detection and classification in turfgrass. Meanwhile, **Table 3** lists other classification models applied in the field of weed detection using deep learning, along with some representative examples of their applications.

**Table 3:** Representative deep-learning-based image classification models for in-field weed detection.

Reference	Target(s)	Classification model(s)	Results
(Alirezazadeh et al., 2024)	Weed species in winter wheat fields from UAV images (Violet, Spergula arvensis, Veronica persica, Chenopodium album, Lamium purpureum)	Deep neural networks with varying complexity (heavy and light CNN architectures), trained using transfer learning	Results indicate that deeper networks failed to effectively learn meaningful feature representations, limiting performance gains for the specific weed classification task; model performance is significantly affected by backbone complexity
(Jin et al., 2025)	Weed grid cells in vegetable fields	Image classification CNNs (EfficientNet, GoogLeNet, ResNet) combined with grid-cell method and image processing	Overall accuracy of EfficientNet, GoogLeNet, and ResNet in identifying vegetable grid cells > 0.956; ResNet achieved highest computational efficiency, 12.76 ms per image, 80.31 fps; effective vegetable identification and weed-soil differentiation improved weed detection accuracy
(Ahmad et al., 2021)	Early-season weeds in corn and soybean fields (Xanthium strumarium, Setaria viridis, Amaranthus retroflexus, Ambrosia trifida)	Image classification models: VGG16, ResNet50, InceptionV3; Object detection model: YOLOv3	Classification: VGG16 best with 98.90% accuracy, 99% F1 score; PyTorch showed faster training and higher accuracy; Detection: YOLOv3 achieved total mAP 54.3% for multi-weed localization and identification; classification suitable for single-weed recognition, detection suitable for SSWM system for multi-weed localization and identification
(Almalky and Ahmed, 2023)	Four growth stages of Consolida regalis weeds in corn fields	Single- and two-stage models: YOLOv5-small/large, RetinaNet (ResNet-101-FPN, ResNet-50-FPN backbone), Faster R-CNN (ResNet-101-DC5, ResNet-101-FPN, ResNet-50-FPN backbone)	YOLOv5-small achieved real-time detection and growth-stage classification with a maximum recall of 0.794; RetinaNet (ResNet-101-FPN) showed test mAP of 87.457%; YOLOv5-large had the highest precision but missed some objects. Overall, RetinaNet (ResNet-101-FPN) demonstrated accurate and high-precision performance.

Deep learning-based weed classification models can automatically learn discriminative features from images through end-to-end training, enabling rapid and high-accuracy weed identification in complex field environments without relying on handcrafted features (Saleh et al., 2024). Moreover, these classification models are capable of distinguishing weeds at different growth stages or of different species, providing valuable support for category-specific precision herbicide application, weed management decisions, and crop yield prediction. Lightweight classification models further allow real-time inference while maintaining high detection accuracy, making them suitable for deployment on UAVs or edge devices. However, these classification models also have inherent limitations. They can only determine the category of an image or sub-image and cannot directly provide precise spatial information on weed locations, which necessitates integration with additional detection or segmentation methods. In addition, high-accuracy deep models typically rely on large-scale annotated datasets, which are costly and time-consuming to acquire. Furthermore, the generalization ability of deep learning models may be limited across different fields, crop types, or varying lighting conditions.

### 2.5 Advantages and limitations of deep learning-based weed detection

Deep learning has enabled significant progress in weed detection by providing three complementary modeling paradigms—classification, object detection, and image segmentation—each offering different levels of spatial and semantic detail. Classification models focus on discriminating weed species or growth stages, object detection models provide bounding-box level spatial localization, and segmentation models deliver pixel-level delineation of weed morphology. Together, these models support a broad range of downstream tasks in precision agriculture, such as targeted herbicide application, weed density mapping, and vegetation monitoring. Their ability to extract hierarchical features directly from raw images enables strong robustness against illumination variation, occlusion, and background complexity in field environments.

Despite these advantages, each model type exhibits inherent limitations that affect its suitability for different application scenarios. Classification models cannot provide explicit spatial localization and therefore require additional mechanisms—such as grid partitioning or region proposals—to determine weed positions. Object detection models suffer from reduced accuracy in dense crop–weed canopies and often miss small weeds, particularly under occlusion or strong illumination variability. Segmentation models achieve the highest spatial precision but incur substantial annotation cost and computational burden, making real-time edge deployment challenging. Selecting an appropriate model thus requires balancing accuracy, spatial detail, computational constraints, and annotation requirements. **Table 4** summarizes the major advantages and limitations of the three model categories.

**Table 4:** Advantages and limitations of three deep learning-based weed detection models.

Model type	Advantages	Limitations
Object Detection	Precise localization of weeds; Able to distinguish different weed species; Output can be directly used for targeted herbicide application and management	Accuracy may decrease in fields with dense crops and weeds or severe occlusion; High computational resource requirements
Segmentation	Pixel-level delineation of weed distribution and morphology; Effectively handles crop occlusion or dense weed growth; Outputs can be directly used for precision spraying, density analysis	High computational complexity; Challenging for real-time deployment on edge devices or UAVs
Classification	Capable of distinguishing different weed species or growth stages	Cannot provide accurate spatial localization of weeds; Limited capability to identify dense weeds under complex field conditions

## 3 Discussion

The fundamental limitation of deep learning-based weed detection does not lie in the network architectures themselves, but rather in the substantial cost and difficulty of constructing high-quality annotated datasets. Although object detection, image segmentation, and image classification models have demonstrated impressive recognition capabilities, all these approaches rely heavily on large quantities of accurately labeled images. In real agricultural environments, however, weeds exhibit extensive species diversity, strong morphological variability, and pronounced spatiotemporal changes across regions, seasons, and growth stages. Meanwhile, pixel-level segmentation requires dense annotations, which are extremely expensive and time-consuming to produce at scale. The long-tail distribution of weed species further aggravates the data imbalance problem, resulting in weak generalization when models encounter rare or unseen species in new environments. Consequently, the dependence on large, meticulously annotated datasets has become one of the major bottlenecks hindering the widespread deployment of deep learning models in real-world precision agriculture.

To alleviate the burden of data annotation, current research has explored two main strategies: indirect weed detection and semi-supervised learning. Indirect weed detection reduces annotation costs by shifting the recognition focus from weeds to crops; only crop images need to be labeled, and non-crop green regions are subsequently treated as weeds (Jin et al., 2022c). While this approach is practical for fields with a single crop species, its performance is constrained by several factors: vegetation extraction using traditional color-based thresholds is highly sensitive to illumination changes; background objects such as soil, straw, and shadows can easily be misclassified as weeds; and most importantly, this method cannot differentiate weed species—an essential requirement for species-specific herbicide application and resistance management. The second strategy, semi-supervised learning, leverages large quantities of unlabeled field images to augment training via pseudo-labels, consistency regularization, or teacher–student frameworks (Kong et al., 2024b). This approach substantially reduces annotation demands and enhances the model’s ability to generalize across regions and seasons. Nevertheless, semi-supervised methods remain susceptible to pseudo-label noise and may struggle with small weeds or heavy occlusion. Overall, indirect detection offers a low-cost entry point for practical deployment, whereas semi-supervised learning provides a promising pathway for long-term, scalable, and region-adaptive weed detection. Future work will likely integrate both strategies to reduce annotation cost while improving robustness in complex agricultural environments.

From an operational perspective, different deep learning models vary substantially in their compatibility with weeding actuators. Object detection and image segmentation models can directly provide spatial information—through bounding boxes or pixel-level masks—which enables seamless integration with sprayers, mechanical cutters, robotic arms, or other weeding mechanisms. For example, precision sprayers can trigger individual nozzles based on the center of a detection box, whereas robotic manipulators can plan motion trajectories according to the contours extracted from segmentation masks (Qi et al., 2023). In contrast, image classification networks output only categorical labels without location information, making them unsuitable for direct actuator control. Despite their advantages in inference speed, compactness, and ease of deployment, classification models alone can only determine the presence of weeds at the image level. To address this limitation, Jin et al. (2022) proposed a grid-based classification strategy in which field images are divided into multiple spatial cells, and each cell is independently classified to approximate weed locations. This approach retains the computational efficiency of classification networks while providing coarse spatial cues, making it suitable for real-time or low-power applications where only approximate localization is required.

In practical deployment, the choice of deep learning model is further constrained by hardware capabilities, communication delays, platform dynamics, and actuator response characteristics. High-precision segmentation models often require substantial computational resources, making real-time inference on edge devices—such as UAVs, autonomous weeding robots, or tractor-mounted terminals—challenging. Lightweight detection or classification models are better suited for devices with limited compute budgets, although they may offer reduced recognition accuracy. Consequently, actual system design must balance accuracy, inference speed, power consumption, and hardware cost while also accounting for vehicle speed, nozzle response time, mechanical inertia, and allowable control latency. Moreover, integration between perception models and weeding actuators often requires intermediate modules for spatial coordinate transformation, temporal synchronization, and delay compensation—for instance, mapping image coordinates to spraying coordinates, predicting future weed positions based on vehicle motion, or using real-time depth maps to adjust operation height. With the ongoing advancement of embedded AI accelerators, model compression techniques, and edge computing, future intelligent weeding systems are expected to become more lightweight, modular, and real-time, enabling a complete pipeline that evolves from “recognition–localization–actuation” toward “prediction–planning–closed-loop control”.

From the perspective of weed management strategies, future weeding approaches are expected to adopt a multi-strategy integration. In inter-row areas, where weed density is relatively low, automated mechanical weeding can be effectively applied. Conversely, in intra-row areas, mechanical weeding may damage crops; therefore, site-specific chemical weeding using robotic systems based on deep learning–driven weed localization is recommended. This approach not only protects crops but also reduces herbicide usage, minimizing potential harm to both crops and the environment. Furthermore, with the rapid development of low-altitude aerial technologies, future strategies may combine UAV-based image acquisition to determine weed locations, generate prescription maps, and guide ground-based weeding robots for precise spot treatment. Such an integrated approach is anticipated to provide a more efficient and intelligent solution for precision agriculture.

In summary, the future of weed management is expected to advance toward a diversified, intelligent, and precision-integrated approach. By combining mechanical, chemical, and deep learning-based intelligent localization techniques, optimal weed control strategies can be selected according to the spatial distribution of weeds and the growth status of crops in different field zones. This enables the efficient removal of weeds while minimizing damage to crops and the surrounding environment. Such multi-strategy, intelligent weed management approaches not only enhance agricultural productivity but also promote sustainable agricultural practices, supporting the development of eco-friendly and high-efficiency precision farming.

## 4 Conclusion

Deep learning has emerged as a powerful foundation for modern weed detection, enabling substantial improvements in robustness, accuracy, and automation compared with traditional machine vision. This review synthesized recent advances across three major model families—object detection, image segmentation, and image classification—and highlighted their respective strengths in spatial localization, pixel-level delineation, and lightweight deployment. Despite this progress, practical adoption remains constrained by the high cost of constructing diverse annotated datasets, limited cross-region generalization, and the computational demands of real-time inference on edge devices. Promising future directions include data-efficient learning strategies (e.g., semi-supervised and domain-adaptive methods), perception–actuation co-design for tighter integration with mechanical and chemical weeding systems, and multi-strategy intelligent weed management that combines deep learning, UAV sensing, precision spraying, and robotic execution. Collectively, these developments point toward a future in which deep learning enables more scalable, sustainable, and precision-integrated weed control across diverse agricultural environments.
